# Psychiatry

**Published:** 1905-04-08

**Authors:** 


### Progress in Medicine and Surgery.

### PSYCHIATRY.

(*Continued from page* 11.)

**Paranoia and its Relation to Modern Witchcraft.** Dr. Conolly Norman, of Dublin, deals with interesting cases of the insanity known as paranoia, and of the strange relations it holds to witchcraft in the popular mind. "Among the sane or partially sane or those usually deemed sane, the secular belief in witchcraft is perhaps the most remarkable example of a combination of feelings—the sense of mystery and the emotion of suspicion." Many of the phenomena of the belief in witchcraft are the same over all the world, and the machinations of the witchfinder among the Matabele are essentially identical with those of the notorious Matthew Hopkins and of the Salem worthies; nor were the "reasons" which convinced the Bishop of Rouen that Jeanne D'Arc was a witch very different from those which satisfied Baxter, the Rev. Cotton Mather, Sir Matthew Hale, Chief Justice of England, or (of worse example) Sir Thomas Browne, the last of whom, merging his belief in the maxim of Tertullian, said of witchcraft, *Certum est quia impossibile est.* One of the most frequent means whereby the witches of old were believed to influence their victims injuriously was to acquire possession of something pertaining to the latter, and by exercising malign influence upon this object, the evil was supposed to be transferred to the bewitched person. Thus waxen images of individuals were used, and pins were thrust into them or the image was slowly melted away, so that by these means pain and disease, or loss of flesh and pining away of the intended victims might be produced. Incantations and blasting spells were added to give these ceremonies their special efficacy and personal application. If the handwriting or a lock of hair of the victim could be obtained, the influence of the witch for harm was supposed to be even greater.

We find, says Dr. Norman, this thought reproduced with singular fidelity, though in a modern garb, among those paranoiacs of to-day who entertain the very common delusions of mystic influence. The following two cases illustrate the matter, and exemplify a curious morbid "reversion" of mind present in such patients. The first was a female aged 20 years, whose mental symptoms appeared three months previously. She had been adopted as an orphan, and at the age of 16 she ran away with her mistress's coachman, leaving him shortly after, owing to his infidelities with other women, and returning to her home in Ireland. She then (January 1902) began to see visions of the Virgin Mary in the workhouse chapel, and had also seen a cloud come down from the ceiling and a cross come out of the cloud. A voice in her ear had directed her to cut off her hair, and she had done so. Attempts were made, she said, in the workhouse to "mesmerise" her. She had numerous other delusions of mystic and malign influence—*e.g.* the asylum officials were going to do something to her, and she knew this because she felt hot when they came near her. "Lights" in the sky appeared, she said, to mesmerise her, and a "voice" told her not to speak, so that "she remained dumb for a considerable time"—which was true. She also heard one of the medical officers "planning with a couple of nurses to mesmerise her." Her diet was "horse flesh"—she knew it, she said, by its peculiar taste—and there was "chloroform" in her tea and on her meat—she knew it by the smell (February and March, 1902). Chloroform was also administered by her persecutors at night through a trapdoor in the ceiling. The medical officers "smelt of chloroform" as they passed her by. She tells the nurse that she (the patient) is the "Duchess of Devonshire," "the Spanish Duchess," "was born in Spain," and that the Blessed Virgin Mary spoke to her and foretold her many things that have come to pass (April-June, 1902). She will eventually, she said, triumph over her persecutors, put down the devilish work of doctors and nurses, and rule the asylum. She dresses in a Spanish peasant costume. She says she is married to one of the doctors, who is a "Spaniard, and about to be a prince." She writes this gentleman love-letters. Early in 1903 she began to accuse Dr. Norman of "influencing" her by mesmeric means. One day she said he stood behind her, and in consequence of a sensation she experienced she turned round and saw him thrust his hand in the tail-pocket of his coat. Ever after she experienced a sharp pain in the back of her chest on the left side whenever she saw him. She complained of "mitral valve disease," and said that Dr. Norman by pointing at her had caused her dreadful pain, and so influenced her heart that she fainted three times next night. Owing to his fatal influence she felt ill whenever she saw him, and had pain in her heart, and sometimes "pain all over as if prodded with pins." His mesmeric influence had "placed a wire in her throat" and caused a feeling "like a reel winding up in her chest, it made her think thoughts she should not think of day and night, and for months she remained dumb for the same reason." Questioned as to how he got such influence over her she replied, "He asked somebody to get my handwriting. I wrote a letter to a girl, who is a friend of mine. They got it and gave it to him, *and once he had my writing and my signature he could influence me by that* as he wished, and from this came all that followed. I was then in his power, he could do what he liked when he had my signature to work on." I have not been able to trace any source from which she can have picked up this notion, says Dr. Norman. It is a perfect reversion to an old witchcraft idea of the peasants, which looks odd in its association with the modern jargon of "loss of will-power," "hypnotism," "mesmerism," etc., but harmonises remarkably well with the mental tone of hallucinatory paranoia—full of suspicion, full of mystery, and possessed by that curious hallucinatory sense of having the thoughts and ideas controlled by some agency outside the patient's eye.

In another case, that of a male patient aged 39 years, and of a family tainted with insanity, the early symptoms were those of "hostility of the public," and "voices" of a woman which kept him awake at night though he did not know her. Even the horses' hoofs had the "power of calling out his name" under the bidding of "electric power." "Flashlights" acted upon him, and they had the power of warning him by bells of impending danger and of transmitting his thoughts to other places. All his trouble he says arose through the phonograph. "Some years ago when the phonograph was new I was asked to speak into it that I might hear my own voice. *I did so, and they have got hold of my phonogram and are always interfering with it. I was sent here because my phonogram was exposed to the public and everybody could work upon it.*" Here again, says Dr. Norman, that very personal thing, "a man's phonogram," plays the part which the autograph signature played in the first case. In both cases we have so striking a coincidence with the machinery of witchcraft that it is difficult to believe that the resemblance is accidental. Such a belief in malign agencies is present in cases of paranoia, and probably many of the witches burnt in times gone by were insane paranoiacs who spoke of these strange agencies in their confessions. That reputedly sane people believed in such utterances and destroyed such "witches" shows that they were really the blind instruments of that hatred of the mentally unsound which seems as natural to man as the instinct which bid gregarious animals destroy one of a herd who is diseased.

**Lesions of the Fundus Oculi in General Paralysis.** Raviart and Caudron state the results of re-examination of 23 cases of general paralysis, the survivors of an earlier series of 51 cases previously examined. Of these, the ones in whom the disease (paresis) had progressed little or not at all remained in practically the same condition as regards the fundus oculi (optic atrophy following choked disc), while in those in whom the disease had progressed the ocular changes were also more pronounced. A further new series of 44 paretics were also examined. Of these 38 showed more or less marked neuritic and atrophic changes in the optic papilla. These changes varied from slight pallor (incipient atrophy) to extreme atrophy of the white or gray type. In five cases the retina showed when examined microscopically after death, a marked increase of the connective tissue and neuroglia elements, this hypertrophy being more conspicuous the greater the atrophy of the nerve-elements proper.

**Simple Idiocy Compared with that of Infantile Cerebral Palsy.**—Dr. W. König of Berlin states [5] that in a previous publication he had shown "that there exists an unbroken chain of cases between infantile cerebral palsy with normal mental condition on the one hand and simple idiocy on the other." That the etiological factors in these two groups of cases are largely identical, the present article is intended to demonstrate. With regard to the cerebral palsy of infants he recognises three certain directly acting etiological factors—namely, (*a*) difficult labour or asphyxia of the fœtus; (*b*) cranial and cerebral traumatism; and (*c*) the infectious diseases of infancy. All other factors are to be considered as predisposing or contributory causes. Dr. König carefully investigated 260 cases of idiocy which upon analysis furnished the following figures, as compared with the similar figures for cerebral palsy.

| *Etiological Factors.* | *Cerebral Palsy.* Per cent. | *Idiocy.* Per cent. |
|---|---|---|
| 1. Insanity or nervous disease in the ancestry ... ... ... ... | 28·5 | 32 |
| 2. Tuberculosis in the ancestry ... | 14·4 | 13·8 |
| 3. Alcoholism in the father ... ... | 23·0 | 15·0 |
| 4. Mental shock or trauma to the mother in pregnancy ... ... | 23·0 | 12·5 |
| 5. Bodily trauma to the mother in pregnancy ... ... ... ... | 2·9 | 3·0 |
| 6. First labour ... ... ... ... | 27·1 | 17·6 |
| 7. Premature labour ... ... ... | 10·0 | 3·8 |
| 8. Illegitimacy ... ... ... ... | 10·0 | 6·5 |
| 9. Child delicate from birth ... ... | 15·7 | 10·0 |
| 10. Child of elderly parents, or the last of a large family ... ... ... | 10·0 | 16·9 |
| 11. Collateral insanity in other children of the family... ... ... ... | 7·1 | 30·7 |
| 12. Collateral tuberculosis in other children of the family ... ... ... | 5·7 | 2·3 |
| 13. Difficult labour or asphyxia ... ... | 11·4 | 10·0 |
| 14. Infectious diseases ... ... ... | 7·1 | 3·4 |
| 15. Syphilis ... ... ... ... ... | 5·5 | 10·0 |

Special attention may be drawn to the fact that insanity, nervous disorder, and alcoholism occur in the ancestry in 47 per cent. of cases of simple idiocy as compared with 51 per cent. of cerebral palsy, while tuberculosis of ancestry occurs in 14 per cent. of each. Difficult labour or asphyxia accounts for 10 per cent. of idiocy and $11\frac{1}{2}$ per cent. of cerebral palsy. Syphilis occurs in the parentage of 10 per cent. of idiots, but only $5\frac{1}{2}$ per cent. of cases of cerebral palsy. Collateral insanity in brothers and sisters, a valuable indication of the family taint of insanity, occurs in 31 per cent. of idiots, whereas it is small (7 per cent.) in cases of cerebral palsy. A critical survey of the facts and figures tend to show that while idiocy and cerebral palsy may show many superficial pathological affinities of causation, that an insane heritage produces twice as much idiocy as cerebral palsy, that alcoholic ancestors beget more palsied than idiotic cases in the proportion of three to two, and that tuberculosis produces equal numbers; while syphilis produced two idiots to one palsied child.

[4] Journ. de Neurologie, 1904, No. 3. [5] Allgem. Zeitschr. für Psychiatrie, 1904, Part 2.

